# Lipoprotein(a) in children and adolescents with genetically confirmed familial hypercholesterolemia followed up at a specialized lipid clinic

**DOI:** 10.1016/j.athplu.2024.06.002

**Published:** 2024-06-20

**Authors:** Anja K. Johansen, Martin P. Bogsrud, Magne Thoresen, Jacob J. Christensen, Ingunn Narverud, Gisle Langslet, Tone Svilaas, Kjetil Retterstøl, Kirsten B. Holven

**Affiliations:** a Norwegian National Advisory Unit on Familial Hypercholesterolemia, Oslo University Hospital, Oslo, Norway; b Unit for Cardiac and Cardiovascular Genetics, Oslo University Hospital, Oslo, Norway; c Department of Nutrition, Institute of Basic Medical Sciences, University of Oslo, Oslo, Norway; d Department of Biostatistics, Institute of Basic Medical Sciences, University of Oslo, Oslo, Norway; e Lipid Clinic, Oslo University Hospital, Oslo, Norway

**Keywords:** Familial hypercholesterolemia, lipoprotein(a), Children, Cardiovascular disease

## Abstract

**Background and aim:**

Many children with an FH mutation also exhibit elevated lipoprotein(a) levels, which is an independent risk factor for atherosclerotic cardiovascular disease. Studies have reported higher levels of lipoprotein(a) in adult and middle-aged women than men. There is limited knowledge on the concentration and change of lipoprotein(a) levels in children with genetic FH, and therefore we investigated sex-differences in lipoprotein(a) level and change in lipoprotein(a) in girls and boys with genetically confirmed FH.

**Methods:**

Medical records were reviewed retrospectively in 438 subjects with heterozygous FH that started follow-up below the age of 19 years at the Lipid Clinic, Oslo University Hospital in Norway, and of these we included 386 subjects with at least one Lp(a) measurement.

**Results:**

Mean (SD) age at baseline was 13.8 (7.3) years and the age was similar between sexes. Girls had a higher lipoprotein(a) level than boys at baseline: median (25–75 percentile) 223 (108–487) vs. 154 (78–360) mg/L, respectively (*p* < 0.01). From baseline to follow-up measurement (mean [SD] 8.9 [6.1] years apart), the mean (95 % CI) absolute and percentage change in Lp(a) level in girls was 151.4 (54.9–247.8) mg/L and 44.8 (16.4–73.1) %, respectively, and in boys it was 66.8 (22.9–110.8) mg/L and 50.5 (8.8–92.3) %, respectively (both p > 0.05).

**Conclusions:**

We found an increase in Lp(a) levels in children with genetic FH with age, and higher levels in girls than boys, which could impact risk assessment and future ASCVD. Further research is needed to elucidate whether subjects with FH could benefit from lipoprotein(*a*)-lowering therapies that are under current investigations.

## Highlights

• Lipoprotein(a) levels were elevated in approximately 24 % of girls and 17 % of boys with genetically confirmed FH.
• Girls had higher baseline Lp(a) levels than boys.
• From baseline to follow-up measurement (mean 8.9 years apart), the mean change in Lp(a) level was a 48 % increase.
• Nearly 1 out of 3 children had an increase in Lp(a) level over 50 % between measurements.
• Three percent of children had high-risk phenotypes with elevated Lp(a) and LDL-cholesterol over the 90th percentile.

## Graphical abstract

**Figure:**
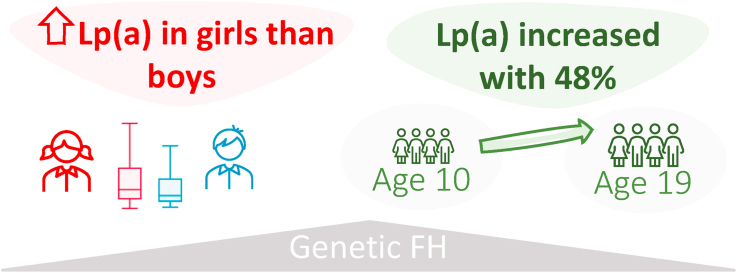

## 1 Introduction

Lipoprotein(a) [Lp(a)] is a plasma lipoprotein consisting of a low-density lipoprotein (LDL) particle to which apolipoprotein(a) is covalently bound, and the levels are mainly genetically determined [1,2]. Higher levels of Lp(a) are associated with increased risk of premature atherosclerotic cardiovascular disease (ASCVD) [[2], [3], [4]] attributed partly to pro-atherosclerotic and pro-inflammatory properties [3,5].

Familial hypercholesterolemia (FH) is an autosomal dominant condition caused by mutations in the LDL-receptor gene (*LDLR*), the apolipoprotein B gene (*APOB*) or the proprotein convertase subtilisin/kexin type 9 gene (*PCSK9*), resulting in elevated LDL-cholesterol from the first year of life, a high life-long exposure to LDL-cholesterol, and high risk of premature cardiovascular disease [6,7]. Having both FH and elevated Lp(a) levels is associated with an even higher risk of ASCVD compared to individuals with only one of these genetic traits [8,9]. Early initiation of lipid-lowering treatment in children with FH, with statins as first drug of choice, is recommended to reduce the life-long LDL-cholesterol burden and ASCVD risk [10]. Effective therapies to lower Lp(a) levels are not yet available, but are currently in development [11,12]. Guidelines from the European Society of Cardiology (ESC) and European Atherosclerosis Society (EAS) recommend measurement of Lp(a) at least once in an individual's lifetime in order to identify and manage those with a higher ASCVD risk [5,13]. Measurement of Lp(a) is specifically recommended for risk evaluation in individuals diagnosed with FH [[3], [4], [5]]. However, variation in Lp(a) levels over time within individuals may be a clinically relevant problem in estimating risk and deciding when to measure [[14], [15], [16]]. In 2740 children with dyslipidaemia visiting a paediatric lipid clinic, 68 % showed a change of at least 20 % between two measurements, with a mean time between first and last measurement of 4.2 years [14]. In contrast, a large prospective multicenter study from Finland involving 3181 participants from the general population with multiple Lp(a) measurements from youth to mid-adulthood suggest more stable concentrations [17]. In these studies, Lp(a) has been measured in either adults or in children and youth with or without dyslipidaemia, but data on Lp(a) levels in children with a genetically confirmed FH are scarce. Interestingly, recent studies have reported higher levels of Lp(a) in adult and middle-aged women than in men [[18], [19], [20]]. Higher Lp(a) levels in girls compared to boys with FH could influence risk evaluation and treatment decisions, however, it is unclear whether sex-differences in Lp(a) is present in these subjects. Therefore, we firstly investigated differences in Lp(a) levels between girls and boys with FH, and secondly, whether Lp(a) levels changed between two Lp(a) measurements during childhood, adolescents, and early adulthood.

## 2 Patients and methods

### 2.1 Study population and design

We retrospectively reviewed the medical records of 438 children and young adults with heterozygous FH that were below 19 years when they started follow-up (between year 1990 and 2010) at the Lipid Clinic, Oslo University Hospital (OUS) in Norway, as described previously [21]. The study complies with the Declaration of Helsinki and was approved by the Regional committee for medical and health research ethics, South-east Norway, as previously described [21,22]. Familial hypercholesterolemia was confirmed by genetic testing in 99 % of the subjects and the remaining were diagnosed clinically based on the Dutch Lipid Clinic Network (DLCN) score >8. Medical record data were collected up to the end of July 2019, where information on age, use of statin medication, height, weight, lipid profile and other blood parameters were extracted.

Of the 438 subjects with medical records reviewed, we included 386 (189 girls and 197 boys) with at least one Lp(a) measurement, where the first (if several measurements) was defined as baseline measurement. Fasting blood samples were analyzed at the Department of medical biochemistry at OUS or at Fürst Medical Laboratory in Oslo. Before year 2014, laboratory assays used to determine Lp(a) concentrations were mass-based and concentrations were therefore reported in mg/L (85.7 % of all values; those collected in the period 1990–2013), and from year 2014 (14.3 % of all values; those collected in the period 2014–2019), both mass-based and molar-based assays were used and reported in mg/L or nmol/L depending on assay. To describe how many children that had elevated levels of Lp(a), we used cut-offs above 500 mg/L and 125 nmol/L, that are often used in clinical settings to define a level that associates with increased risk of CVD. Since most Lp(a) levels were reported in mg/L at baseline (n = 337: 87.3 %), we converted levels in nmol/L to mg/L (49/386: 12.7 %), using Lp(a) (mg/dl) = Lp(a) (nmol/l) * 0,4167 described by Scharnagl et al. [23], in order to compare Lp(a) level between sexes at baseline.

To investigate change in Lp(a) level between two time points (baseline and latest follow-up measurement), we compared only non-converted levels where all levels were analyzed using a mass-based assay (reported in mg/L). We excluded those that were pregnant or lactating at measurement (n = 2).

### 2.2 Statistical analysis

Statistical analyses were performed using Stata (version 18). For Lp(a) levels that were reported below a given detection limit (13.8 % of all values), the value was set to the exact detection limit (e.g. <10 was set to 10) and when Lp(a) levels were reported below different detection limits at first and second time point (e.g < 1 mg/L at baseline and <20 mg/L at follow-up), both values were set to the lowest detection limit (n = 3/70). All tests were two-sided and a significance level of 5 % was set. Baseline characteristics were compared across sexes using Independent samples *t*-test for normally distributed data and Mann-Whitney *U* test for non-normally disitrbuted data, and Chi-Square test for categorical data. Differences between two time points on dependent samples were tested using paired samples *t*-test for normally distributed data, Wilcoxon signed-rank test for non-normally distributed data, and McNemar's test for proportions. Data are given as mean (SD), unless otherwise stated.

## 3 Results

### 3.1 Baseline characteristics

Most subjects were <18 years of age at baseline Lp(a) measurement (n = 300/386:77.7 %). Mean age at baseline was 13.8 (7.3) years and there was no difference in age between girls and boys (*p* > 0.05, Table 1). Ninety-nine percent (382/386: 99 %) had a genetically verified FH mutation (Table 1), either in the *LDLR* (374/382: 98 %) or the *APOB* (8/382: 2 %, data not shown in Table). Two-hundred and eight (54 %; 111 boys and 97 girls) had an LDL-receptor null mutation, and the proportion did not differ between the sexes (*p* = 0.33, data not shown in Table). There was no difference in baseline Lp(a) level between those with and without a null mutation in the *LDLR* (*p* = 0.98). At baseline, 19.9 % of subjects were using statins (Table 1); compared to non-statin users, statin users were older, with mean age 24.1 (7.68) years vs. 11.2 (4.0) years at baseline, respectively (*p* < 0.001, data not shown). The percentage of girls using statins at baseline was 16.4 % compared to 23.4 % of the boys (*p* = 0.09, Table 1), and although there was no statistically significant difference in age at statin start between girls and boys, there was a tendency towards higher age at statin start among girls compared to boys, with mean age 15.3 (3.8) years in girls and 14.6 (3.7) years in boys (*p* = 0.23). Sixty-seven (17.3 %) had an FH parent with premature CVD (<51 years for men and <56 years for women), with no difference between parents of either sex (*p* = 0.52). Median (25–75 percentile) baseline Lp(a) level was 253 (95–526) mg/L and 179 (87–404) mg/L in those with and without premature CVD in FH parent (*p* = 0.18), respectively. For girls and boys with premature CVD in their FH parent, the median (25–75 percentile) Lp(a) level at baseline was 411 (114–571) mg/L in girls and 189 (83–344) mg/L in boys (*p* < 0.05). Subjects with reported premature CVD in their FH parent were older than those without, respectively 15.5 years versus 13.5 years (*p* = 0.04).

**Table 1:** Baseline characteristics.

	All	Girls	Boys	*p-*value
Number of patients	386	189	197	
Age (years)	13.8 (7.3)	13.9 (7.7)	13.8 (6.8)	0.82
Lp(a) (mg/L)^a^^,^^b^^,^^c^	191 (93–419)	223 (108–487)	154 (78–360)	**<0.01**
Lp(a) > 500 mg/L or >125 nmol/L, n (%)^c^	78 (20.3)	45 (24.1)	33 (16.7)	0.07
Statin medication, n (%)	77 (19.9)	31 (16.4)	46 (23.4)	0.09
Genetically verified FH, n (%)	382 (99.0)	188 (99.5)	194 (98.5)	0.33
LDL-cholesterol (mmol/L)	5.5 (1.9)	5.8 (2.0)	5.2 (1.7)	**<0.01**
Total cholesterol (mmol/L)^d^	7.3 (1.9)	7.6 (2.0)	7.0 (1.8)	**<0.01**
HDL-cholesterol (mmol/L)^e^	1.4 (1.1)	1.4 (1.0)	1.4 (1.3)	0.92
TG (mmol/L)^a^^,^^f^	0.80 (0.60–1.10)	0.80 (0.60–1.05)	0.70 (0.50–1.10)	0.11

Data are shown as mean (standard deviation), unless otherwise stated. Abbreviations: HDL: high-density lipoprotein; LDL: low-density lipoprotein, TG: triglycerides, Lp(a): lipoprotein(a). Differences between sexes were tested by a Two-Sample *t*-test for normally distributed variables, and a two-sample Wilcoxon rank-sum (Mann-Whitney) test for non-normally distributed variables. A Chi-squared test was used to test the distribution of categorical variables between sexes.

a Stated as median (25–75 percentile).

b Levels measured in nmol/L were converted to mg/L in 49 subjects (12.7 %).

c Two subjects were pregnant/lactating at measurement and not included in Lp(a) level analysis.

d Total cholesterol level missing in 2 girls.

e HDL-cholesterol level missing in 1 girl.

f Triglyceride level missing in 2 girls and 2 boys.

### 3.2 Elevated Lp(a) in boys and girls with genetically confirmed FH

**Fig. 1:**
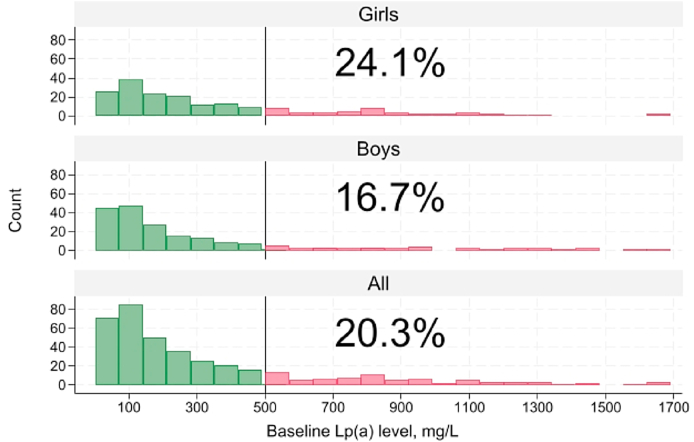
Distribution of baseline Lp(a) level in 187 girls and 197 boys, and percentage of patients with elevated level (>500 mg/L). Levels measured in nmol/L were converted to mg/L (n = 49/386: 12.7 %). Two female subjects excluded from analysis due to pregnancy/lactation.

**Fig. 2:**
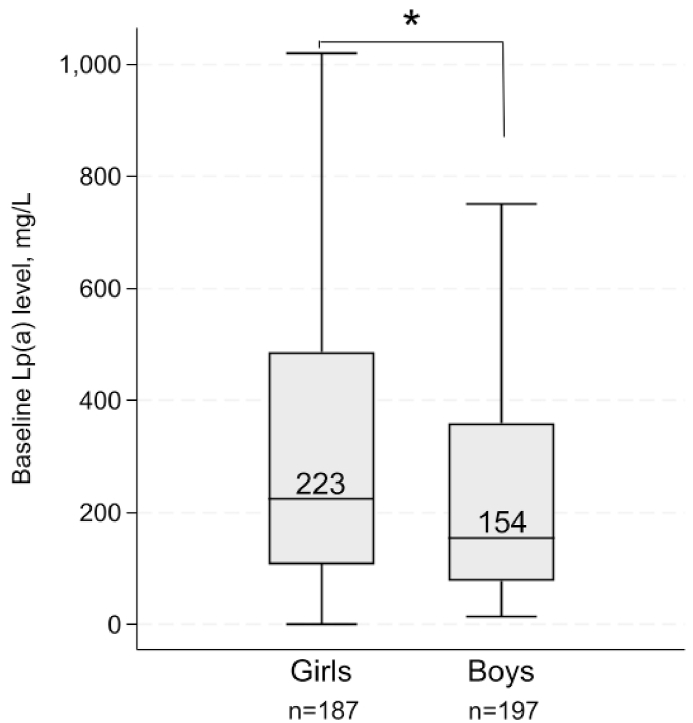
Boxplot of baseline Lp(a) level in girls and boys. Two female subjects were excluded due to pregnancy/lactation. Differences between sexes were tested by a two-sample Wilcoxon rank-sum (Mann-Whitney) test. *p < 0.01. Outliers are not shown.

Approximately 24 % of the girls and 17 % of the boys had elevated Lp(a) levels above the clinical cut-offs (Table 1, Fig. 1). Girls had significantly higher Lp(a) levels compared to boys at baseline, shown in Fig. 2 (the same results are also seen for patients <18 years at baseline, shown in Supplementary Fig. 1). Total cholesterol and LDL-cholesterol levels were also significantly higher in girls compared to boys at baseline (Table 1, all *p* < 0.01). Among children (<18 years at baseline), 9 out of 300 (3 %) had a particular unfavorable high-risk phenotype with an Lp(a) level >500 mg/L or >125 nmol/L, and an LDL-cholesterol level >the 90th percentile (corresponding to an LDL-cholesterol level >7.9 mmol/L). These high-risk children were between 6 and 14 years of age, and 89 % of them (n = 8/9) were girls.

### 3.3 Increase in Lp(a) between measurements

**Fig. 3:**
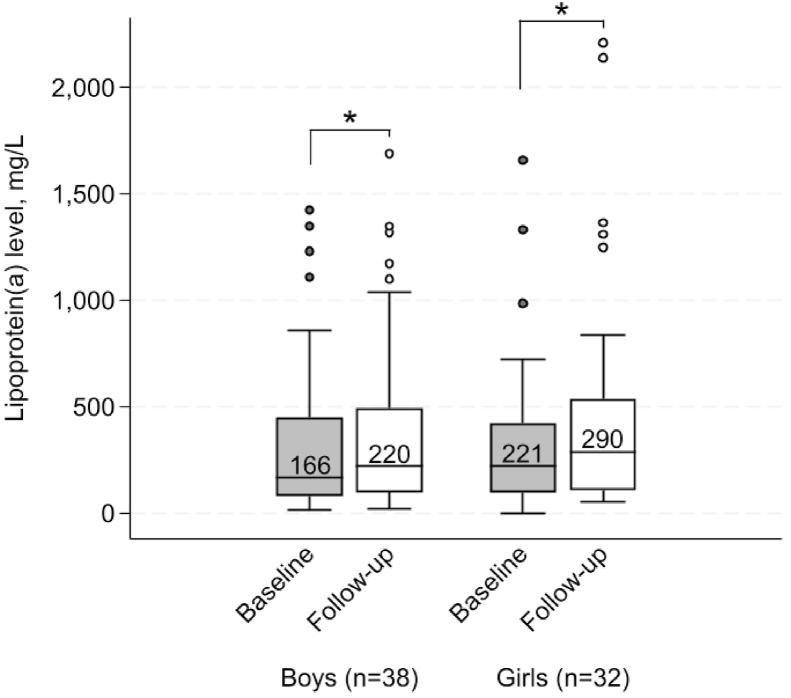
Baseline and follow-up Lp(a) level in boys and girls. Two girls excluded due to pregnancy/lactation. Difference between baseline and follow-up Lp(a) on dependent samples were tested using Wilcoxon signed-rank test for non-normally distributed data. *p < 0.001.

For those with at least two measurements of Lp(a) reported in mg/L (70/386: 18.1 % of subjects) we compared age, blood lipids and statin medication between the first (baseline) and the latest (follow-up) time point (shown in Table 2). In the same group of subjects, the median Lp(a) level increased with 30 % from baseline to follow-up, from median 185 mg/L to 240 mg/L, respectively (Table 2). In both sexes, Lp(a) levels increased significantly from baseline to follow-up (both *p* < 0.05, Fig. 3**).** The mean time between measurements was 8.9 (6.1) years (ranging from 1 to 25 years), and there was no difference between girls and boys, nor the age at either time points (all *p* > 0.05). Most children (97.2 %) did not use statins at first measurement, and at second measurement, 61 % of children were using statins (and no one were using PCSK9-inhibitor), and as Lp(a) levels increased within subjects between measurements, LDL-cholesterol decreased significantly in the same period (*p* < 0.001, Table 2). Among statin users, there was no difference in age between sexes (*p* > 0.05) and the percentage of girls and boys that used statins were the same (66 % of girls and 58 % of boys, *p* > 0.05). Lipoprotein(a) level changed significantly from baseline to follow-up in both children that started statins and those that did not, from median (25–75 percentile) 211 (99–473) mg/L at baseline to 292 (103–666) mg/L at follow-up in statin-users (*p* < 0.005 for difference between baseline and follow-up levels on dependent sample), and 111 (59–374) mg/L at baseline to 153 (80–485) mg/L at follow-up in non-statin users (*p* < 0.05 for difference between baseline and follow-up levels on dependent sample). The mean (95 % confidence interval [CI]) absolute change in Lp(a) level from baseline to follow-up was not statistically different between those that started statins and those that did not, although the increase tended to be higher in statin-users than non-statin users (mean 142.2 [64.7–219.6] mg/L versus 45.2 [86–81.8] mg/L, respectively, *p* = 0.054). Furthermore, statin-users were older than non-statin users at follow-up measurement, with mean age 23.4 (8.6) years versus 14.1 (4.6) years, respectively (*p* < 0.001), and the time between measurements was significantly larger (mean 11.7 [5.8] years in statin-users versus 4.5 [3.1] years in non-statin users, *p* < 0.001). The percentage increase in Lp(a) level was also similar between statin-users and non-statin users (mean [95 % CI] 45.9 [21.6–70.2] % in statin-users versus 51.0 [−5.3-107.3] % in non-statin users, *p* > 0.05). For girls, the absolute and percentage change in Lp(a) level was mean (95 % CI) 151.4 (54.9–247.8) mg/L and 44.8 (16.4–73.1) %, respectively, and for boys it was 66.8 (22.9–110.8) mg/L and 50.5 (8.8–92.3) % (both *p* > 0.05 for difference between sexes). The mean absolute and percentage change in Lp(a) level from baseline to follow-up for those with <5 years, 5 to <10 years, 10 to <15 years and >15 years between baseline and follow-up measurements are shown in Supplementary Table 1.

**Table 2:** Changes from baseline to follow-up.

	Baseline	Follow-up	*p-*value
Number of patients	70	70	
Female gender, n (%)	32 (45.7)	–	
Age (years)	10.6 (4.2)	19.5 (8.3)	
Statin medication, n (%)	2 (2.8)	43 (61.4)	**<0.001**
Lp(a) > 500 mg/L, n (%)	14 (20.0)	19 (27.1)	0.06
Lp(a) (mg/L)^a^	185 (87–449)	240 (100–530)	**<0.001**
LDL-cholesterol (mmol/L)	6.0 (1.6)	4.5 (1.8)	**<0.001**
Total cholesterol (mmol/L)	7.8 (1.7)	6.4 (2.0)	**<0.001**
HDL-cholesterol (mmol/L)	1.3 (0.4)	1.3 (0.4)	0.87
TG (mmol/L)^a^^,^^b^	0.70 (0.50–1.06)	0.80 (0.60–1.20)	0.12

Data are shown as mean (standard deviation), unless otherwise stated. Abbreviations: LDL: low-density lipoprotein, Lp(a): lipoprotein(a). Difference between baseline and follow-up on dependent samples were tested using Paired sample *t*-test for normally distributed data, Wilcoxon signed-rank test for non-normally distributed data, and McNemar's test for proportion data.

a Stated as median (25–75 percentile).

b Triglyceride level missing in 2 subjects at baseline (2.8 %) and 1 subject at follow-up (1.4 %).

### 3.4 Within-subject variation in Lp(a) level

From baseline to follow-up, the overall percentage change in Lp(a) level was a mean (95 % CI) increase of 47.9 (22.4–73.4) %. The Lp(a) level decreased with >20 % in 10 % of children (n = 7/70) and increased with >20 % in 48 % of children (n = 34/70), and moreover, the increase was >50 % in 31 % of children (n = 22/70). Furthermore, 8.6 % of children (n = 6/70, 2 boys and 4 girls) had an increase in Lp(a) level across the clinical cut-off (>500 mg/L) from baseline (mean level was 390 mg/L) to follow-up (mean level was 773 mg/L). On the other hand, of those with baseline levels below 500 mg/L, the great majority (50/56: 89.3 %) had a level below this cut-off also at follow-up. The results were similar when using 300 mg/L as cut-off, thus, of those with lower baseline Lp(a) levels (<300 mg/L), 40 of 45 patients (88.8 %) had lower levels also at follow-up. Furthermore, the proportion of patients with elevated levels >500 mg/L did not change significantly from baseline, whereof 20 % had elevated levels, to follow-up, whereof 27 % had elevated levels (*p* = 0.06, Table 2).

## 4 Discussion

In the present study, we investigated Lp(a) levels in girls and boys with genetically confirmed FH, followed-up at a specialized lipid clinic. In a cohort of children and adolescence with FH that has not been sufficiently described in previous literature, we found that girls have higher Lp(a) levels than boys and that for both sexes, plasma levels increased significantly between two measurements minimum 1 year apart, during childhood and early adulthood (see **Graphical abstract**). For some children the increase was of potential clinical relevance, with 31 % of children having an increase of more than 50 % and 8 % of children increased their Lp(a) level above the threshold suggested for increased risk of CVD. However, most children that had low levels of Lp(a) at baseline, had a low level of Lp(a) also later on.

Our data on sex-differences in children and adolescents with genetically confirmed FH extend previously published data, which mainly comes from adults without FH. In the general population, higher Lp(a) levels are shown in middle-aged women, and in women after menopause, when compared to men [[24], [25], [26]], and similar results has been shown in adult subjects with FH [27]. In children with FH there are fewer studies, however, in children without FH, levels were higher in girls compared to boys [28] and in children with dyslipidemia (68 % FH) there was no difference in Lp(a) level between sexes [14]. Further research is needed to elucidate the mechanisms that could contribute to higher Lp(a) levels seen in females. Different hormonal treatments [29,30] and pregnancy [31,32] are shown to influence Lp(a) levels, which could suggest that differences in hormones may contribute to sex-differences in Lp(a) levels. Genetic factors and non-genetic factors as diet [33] and exercise [34] could also be potential contributors. Statin medication are suggested to increase levels of Lp(a) [35], however, it was not likely a contributing factor to higher Lp(a) levels in girls than boys in our study as only 16 % of girls were using statins and 23 % of boys. Recently, attention has been drawn towards the increase in ASCVD mortality in younger women [36,37], which underlines the importance of our findings since high Lp(a) levels in girls may impact their ASCVD risk.

We found that Lp(a) levels increased during childhood and early adulthood in our assessment of two measurements taken between 1 and 25 years apart. Among children with FH, the mean increase in Lp(a) level was 47 % between, and in those that started statins during this time (60 % of children) it increased with 46 % and with 51 % in non-statin users. These results are partly supported by findings from de Boer et al., that showed a mean increase in Lp(a) level of 43 % in children on statin medication, but the increase was lower (22 %) in children without statin medication [14], however the number of children without lipid-lowering medication in our study was low. Other studies in healthy subjects have also suggested that Lp(a) increases with age, already around the first year of life [38], in children and adolescents [28], and during adulthood [17,24]. Most of the studies mentioned however, are cross-sectional, including only one Lp(a) measurement per person, whereas we assessed two Lp(a) levels within the same subject. We reported that approximately 1 out of 3 children had an increase in Lp(a) level of more than 50 % between first and second measurement taken mean 9 years apart. Importantly, the increase in Lp(a) level seen in 8.6 % of the children in our study, could have implications in clinical care, as their level was initially low, and at a later time point it exceeded the threshold suggested for increased CVD risk. The percentage of subjects with previously lower levels and higher levels (>300 mg/L) at latest measurement was only 3–6% in a previous study of healthy individuals with repeated Lp(a) measures taken between 4 and 25 years apart [17], which could suggest that this is more prevalent among younger subjects with FH. Previous studies have demonstrated that 68 % of children and 40 % of adults had variations in Lp(a) level >20 % between measurements taken 4.2 years apart, and >25 % between measurements taken up to 190 days apart, respectively [14,39]. Clinicians should be aware that larger fluctuations across clinical thresholds could be projected in some children and repeated Lp(a) measurements during childhood could be useful to define risk and decide treatment more precisely, especially in children at high risk like FH with levels between 300 and 500 mg/L (or 75–125 nmol/L). Although Lp(a) levels may increase during childhood to some extent, most of the children in our study, however, had levels within the same risk-thresholds at both time points, which are consistent to previous studies that have shown strong correlations of paired individual blood samples [17,40].

Very limited data exists on Lp(a) levels, especially repeated levels, in children with genetically verified FH. It has previously been shown that the levels of Lp(a) more often is elevated in children with probable FH compared to those with a definite FH diagnosis, suggesting that the elevated Lp(a) levels may be the underlying cause of the probable FH diagnosis [41]. Therefore, measurement of Lp(a) in children suspected for FH is particularly important when no FH-causing mutation is detected. A major strength of our study was that it comprised children and adolescents where an FH diagnosis that was genetically confirmed in 99 % of subjects. To reduce the risk of bias when assessing Lp(a) at two different time points, we assessed Lp(a) levels of subjects with both Lp(a) concentrations measured by the same type of assay (mass-based assays). We cannot however exclude the possibility that some blood samples were analyzed by different types of mass-based assays and/or at different laboratories and therefore some variation in Lp(a) levels can be related to this. In terms of the accuracy of the data, there are some uncertainties that could be addressed. Firstly, mass-based assays are not always reflective of the exact particle number (depending on the number of KIV-2 repeats), which could lead to either over- or underestimations of Lp(a) levels [42]. Secondly, levels that were below detection limits was set to the detection limit before analysis (e.g. <100 mg/L was set to 100 mg/L and <60 mg/L was set to 60 mg/L), which could result in an overestimation of the level in some few cases. Thirdly, a small number of Lp(a) levels (12.7 % of measurements) were converted from nmol/L to mg/L to make the data comparable. However, we assume that this effect is only marginal, and the same results were shown when converted levels were excluded from the analysis. It is important to acknowledge that our findings are not necessarily generalizable to all ethnicities, as the people seen in this specific lipid clinic are mostly native Norwegians. Future investigations of repeated Lp(a) levels in children with genetically confirmed FH require ethnically diverse populations and uniformity of the measurements.

Lipoprotein(a) is an important and independent risk factor for ASCVD and a significant proportion of children with FH have elevated levels. Screening for high Lp(a) in childhood gives the opportunity from early on to influence health by educating our patients and emphasize the importance of a heart-healthy diet, physical activity and preventing tobacco use, in addition to medical treatment [43]. Randomized cardiovascular outcome trials are needed to provide final evidence of causality and to assess potential clinical benefit of novel, potent Lp(a) lowering therapies [44].

## Funding

The study was supported by the South-Eastern Regional Health Authority, Oslo, Norway, the Norwegian National Advisory Unit on FH, Oslo University Hospital, Oslo, Norway, and the Throne-Holst Foundation for Nutrition Research, 10.13039/501100005366University of Oslo, Oslo, Norway and the 10.13039/501100005366University of Oslo, Oslo, Norway.

## Author contributions

**Anja K. Johansen**: Data collection, data analysis, drafting and revision of manuscript. **Martin P. Bogsrud**: Design and planning of the study, interpretation of data, revision of manuscript. **Magne Thoresen**: Statistical analysis and figures, revision of manuscript. **Jacob J. Christensen**: Data analysis, revision of manuscript. **Ingunn Narverud**: Data collection, revision of manuscript. **Gisle Langslet**: Interpretation of data, revision of manuscript. **Kjetil Retterstøl:** Interpretation of data, revision of manuscript. **Kirsten B. Holven**: Design and planning of the study, interpretation of data, revision of manuscript.

## Declaration of competing interest

The authors declare the following financial interests/personal relationships which may be considered as potential competing interests:Dr. Bogsrud has received research grants and/or personal fees from 10.13039/100002429Amgen and 10.13039/100004339Sanofi, none of which are related to the content of this manuscript. Dr. Retterstøl has received research grants and/or personal fees from Akcea, 10.13039/100002429Amgen, 10.13039/100004336Novartis, and 10.13039/100004339Sanofi, none of which are related to the content of this manuscript. 10.13039/501100007212GL reports personal fees from 10.13039/100002429Amgen, 10.13039/100004339Sanofi and Boehringer Ingelheim, none of which are related to the content of this manuscript. Dr. Holven has received research grants and/or personal fees from 10.13039/100004339Sanofi, none of which are related to the content of this manuscript. Msc. Johansen, Dr. Christensen and Dr. Narverud have nothing to disclose.
