# Supplementary material for: Lipoprotein(a) in children and adolescents with genetically confirmed familial hypercholesterolemia followed up at a specialized lipid clinic
Source: Atheroscler Plus. 2024 Jun 20;57:13–8. doi: 10.1016/j.athplu.2024.06.002 (PMC11254952; doi:10.1016/j.athplu.2024.06.002)
Supplement: Multimedia component 1 [file mmc1.docx]

**Supplementary Table 1.** Mean absolute and percentage change in Lp(a) level from baseline to follow-up, stratified by years between measurements.

| Yrs between measurements | N | % females | Mean (SD) age at baseline | Mean (SD) age at follow-up | Mean (SD) absolute change in Lp(a), mg/L | Mean (SD) % change in Lp(a) | Median (25-75 percentile) Lp(a) level at follow-up |
| --- | --- | --- | --- | --- | --- | --- | --- |
| <5 yrs | 21 | 47.6 | 10.0 (3.2) | 12.8 (3.2) | 62 (143) | + 34 (77) | 156 (100-369) |
| 5 to <10 yrs | 19 | 47.4 | 9.6 (3.8) | 15.9 (3.9) | 130 (157) | + 76 (164) | 215 (60-666) |
| 10 to <15 yrs | 15 | 46.7 | 11.3 (3.8) | 22.8 (3.6) | 127 (327) | + 31 (72) | 226 (100-492) |
| ≥15 yrs | 15 | 40.0 | 12.0 (5.9) | 30.3 (8.4) | 114 (203) | + 48 (78) | 373 (164-530) |

Abbreviations: Lp(a): lipoprotein(a), SD: standard deviation, Yrs: years. Two subjects excluded due to pregnancy/lactation (n=2).
