# Supplementary material for: Lipoprotein(a) in children and adolescents with genetically confirmed familial hypercholesterolemia followed up at a specialized lipid clinic
Source: Atheroscler Plus. 2024 Jun 20;57:13–8. doi: 10.1016/j.athplu.2024.06.002 (PMC11254952; doi:10.1016/j.athplu.2024.06.002)
Supplement: Multimedia component 2 [file mmc2.docx]

**
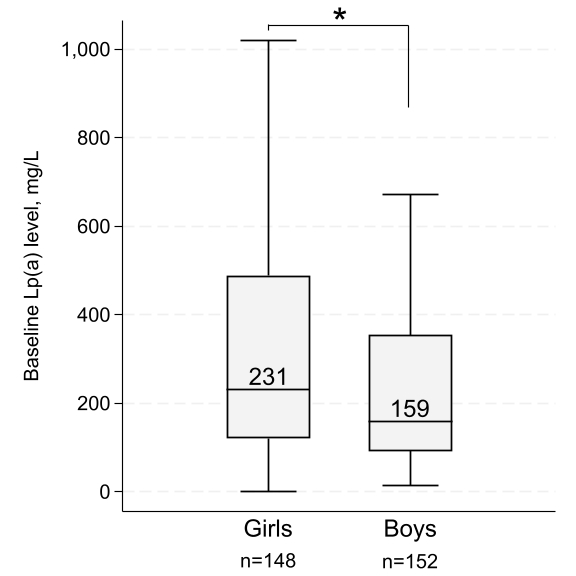
**

**Supplementary Figure 1.** Boxplot of baseline Lp(a) level in girls and boys under 18 years of age. Differences between sexes were tested by a two-sample Wilcoxon rank-sum (Mann-Whitney) test. **p*<0.01. Outliers are not shown.
